# Fertility patients and their prescriptions: a two-year audit of patient-pharmacist interactions in a reproductive endocrinology practice

**DOI:** 10.1186/1755-7682-2-24

**Published:** 2009-08-03

**Authors:** Eric Scott Sills, Serhiy A Shurpyak, Deirdre J Gorman, Lyuda V Shkrobot, Grainne U Murray, Beppi MG O'Connor, Una E Rapple, Alicia O Fogarty, Pavlina Svarkova, Kathy M Brickell, David J Walsh

**Affiliations:** 1 The Sims Institute and Sims International Fertility Clinic, Dublin, Ireland; 2 Department of Obstetrics and Gynecology, School of Medicine, Lviv National Medical University, Lviv, Ukraine

## Abstract

**Background:**

This study assessed pharmacy performance and satisfaction as reported by patients during ovulation induction therapy.

**Materials and methods:**

Patients (*n *= 1269) receiving gonadotropin prescriptions for intrauterine insemination or *in vitro *fertilisation-embryo transfer in 2007–2008 were prospectively interviewed by nurses and/or completed a structured questionnaire to evaluate pharmacy performance. "Community" (*n *= 12) and "specialty" (*n *= 2) pharmacy status (C vs. S) was defined by each pharmacy, and all pharmacies were selected by patients before cycle start. Patient comments about their pharmacy were classified into five types: i) Dispensing error-gonadotropin, ii) Dispensing error-non gonadotropin, iii) Mistake in prescribed medical equipment/supplies, iv) Counselling/communication inaccuracy, and v) Inventory problem or other.

**Results:**

391 pharmacy concerns were reported from 150 fertility patients during the study period. The majority (75.9%) of patients selected a S pharmacy to fill their prescriptions, and this pharmacy type was identified in 2.8% of adverse pharmacy encounters (*p *< 0.0001). Non-gonadotropin prescriptions filled at C pharmacies accounted for 40.2% of all complaints, followed by problems with prescriptions for supplies (20.2%) and gonadotropins (18.7%) at C pharmacies. Patient conflict involving S pharmacies was limited (*n *= 11), and related to operating hours and medication delivery logistics.

**Conclusion:**

Fertility patients reported a disproportionate and significantly higher number of adverse pharmacy encounters from C pharmacies compared to S pharmacies. Although no licensing mechanism in Ireland currently recognises special training or certification in any area of pharmacy practice, informal self-designations by pharmacies remain a useful discriminator. Level of familiarity with fertility medicines and availability of inventory are important characteristics to be considered when counselling fertility patients about pharmacy choice. Those who select a C pharmacy should be advised to allow extra time for inventory verification, order confirmation, and additional counselling. Additional study is needed to determine if a minimum volume of fertility-related prescriptions is necessary to assure competence in this particular field of pharmacy practice.

## Background

Emotional stress associated with infertility (and the pharmaceutical interventions intended to treat it) can be considerable for some patients. Failure of IVF treatment after multiple attempts can be especially devastating for couples [1]. Patient unfamiliarity with gonadotropin self-injection, hormone supplements, and other medicine required for ovulation induction cycles represent important potential stressors of the infertility treatment experience. The occurrence of any adverse pharmacy encounter (including dispensing error) reduces confidence in treatment precision and contributes to increased patient anxiety. While prior research has focused on self-reported physical and/or emotional issues related to fertility treatment [2], there has been no specific evaluation of pharmacy encounters in the infertility stress equation. Developing effective interventions for pharmacists is predicated on an understanding of exact needs from the patient's perspective [3], and pharmacy communication is an important part of fertility treatment. In this investigation, we sought to evaluate patient satisfaction with pharmacy services provided during advanced fertility treatment with a view to estimate the frequency and type of pharmacy problems encountered.

## Methods

This study analysed data from 1,269 advanced fertility treatment cycles at Sims IVF Clinic in Dublin, prospectively gathered during 2007 and 2008. Feedback questionnaires and notes from structured nursing interviews with patients (as entered into the medical record) were reviewed for patients undergoing gonadotropin therapy to evaluate satisfaction with pharmacy services. These data captured patient-generated alerts involving pharmacies involved with dispensing medication for all intrauterine insemination and *in vitro *fertilisation-embryo transfer treatment cycles, irrespective of status as new or return patients.

Charts were assessed for documentation to confirm each patient's pharmacy choice was made independently and prescriptions were tracked to determine which pharmacy that the patient selected to fill her medication order. All communication received from pharmacies (either telephonically or in writing) was also reviewed for the study interval. Pharmacy events reported by patients were assigned to one of the following encounter types: Category I – Dispensing error (gonadotropin), Category II – Dispensing error (non-gonadotropin), Category III – Dispensing error (medical equipment/supplies), Category IV – Counselling or communication inaccuracy, or Category V – Inventory problem or problem not otherwise specified.

Although some fertility patients generated multiple complaints, each issue was separately detailed to facilitate management and analysis; each specific problem or complaint lodged by a fertility patient was recorded only once.

Data on patient satisfaction with pharmacy service were stratified by pharmacy type, either "community" (C) or "specialty" (S). This was an unofficial designation made by the pharmacy itself; in cases where pharmacy status was unknown or uncertain, a member of our nursing staff directly communicated with the relevant pharmacy manager for clarification and subsequent status assignment.

A total of twelve C and two S pharmacies were subjects of comment by fertility patients during the study interval. Pharmacy self-designation status remained unchanged for any retail pharmacy that appeared in both study years. No patient used a combination of the two pharmacy types during any fertility treatment sequence. Records describing patient-pharmacy encounters with pharmacies outside the Republic of Ireland were not considered for assessment. Data were analysed by Student's *t*-test or z-test for proportions, as appropriate.

## Results

**Figure 1:**
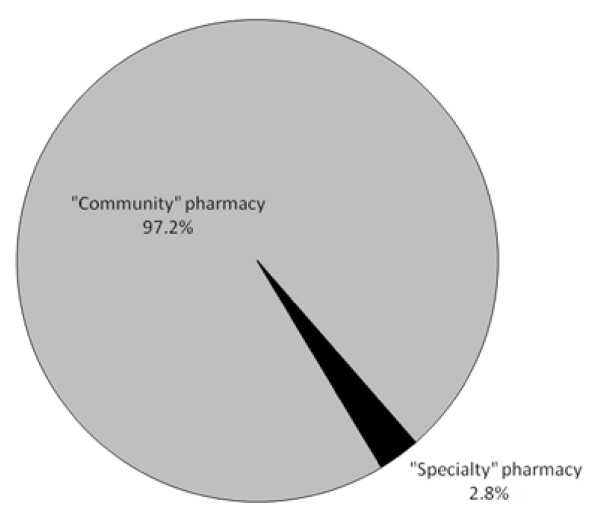
**Source of adverse pharmacy encounter alerts generated by Irish fertility patients during 2007 and 2008, by pharmacy type (*n *= 391)**.

During the two year study period, 75.9% of patients selected an S pharmacy and 24.1% selected a C pharmacy to dispense their medicines (*p *< 0.0001). Our quality management system tracked 391 individual pharmacy complaints or concerns from 150 fertility patients during this interval, corresponding to an overall pharmacy complaint rate of 11.8%. Study patients distributed their prescriptions among 12 C and 2 S pharmacies in 2007 and 2008. C and S pharmacies accounted for 97.2 and 2.8% (*p *< 0.0001) of adverse pharmacy encounters, respectively, as shown in Figure 1.

We sought to identify any possible common features in the smaller S pharmacy complaint group (*n *= 11) first. All eleven adverse encounters in this sub-set involved dissatisfaction with limited office hours, complaints about the inability to provide home or workplace delivery, or other logistical issues that could not be resolved (Type V). No other encounter type involving S pharmacies was reported by study patients.

**Figure 2:**
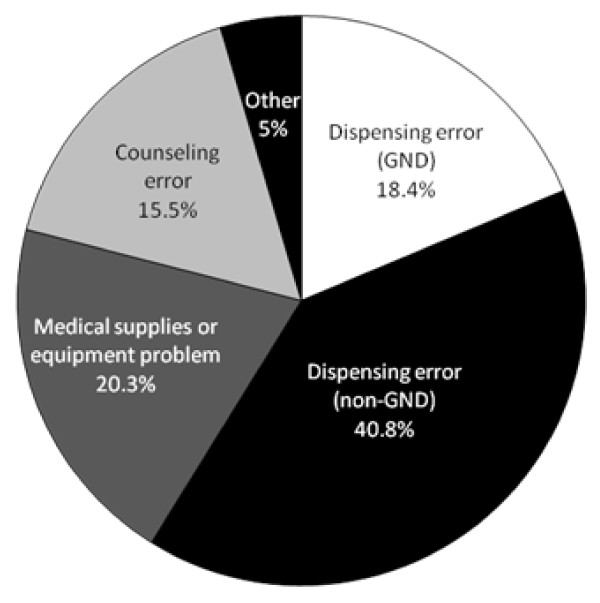
**Distribution of community pharmacy complaints (*n *= 391) by fertility patients**.

The sample deriving from fertility patients who chose a C pharmacy was studied next (see Figure 2). In this group, errors involving non-gonadotropin prescriptions (Type II) accounted for 40.8% of complaints (*n *= 155), including incorrectly filled prescriptions for anti-coagulants, immune modulators, antibiotics, vitamins and/or narcotics. Among our fertility patients, 20.3% of C pharmacy service concerns involved problems with prescribed medical equipment or supplies (Type III), including wrong needle size, insufficient number of syringes dispensed, failure to provide the "safe sharps bin", or in one case, the refusal to dispense any equipment for subcutaneous injection of gonadotropin. In 18.7% of unsatisfactory pharmacy encounters, our fertility patients experienced problems with specific gonadotropin prescriptions (Type I).

**Figure 3:**
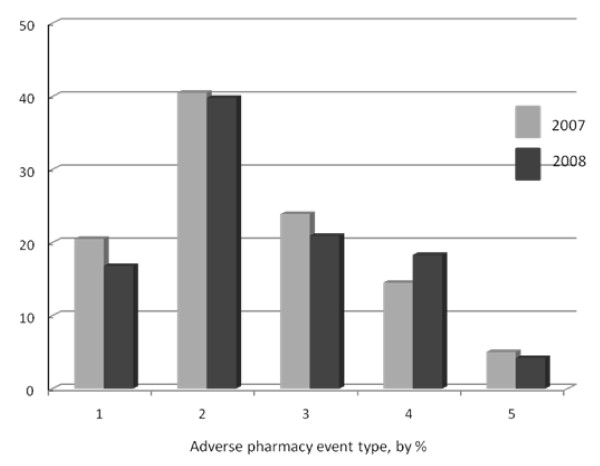
**Frequency of adverse pharmacy encounters reported by fertility patients (*n *= 150) during a two year audit**. Type 1 = Dispensing error-gonadotropins; Type 2 = Dispensing error-non gonadotropin; Type 3 = Dispensing error involving medical equipment/supplies; Type 4 = Counselling/communication error; Type 5 = Inventory problem or not otherwise specified. No significant difference was observed between 2007 and 2008 for any event type (*p *> 0.20, by z-test for proportions, CI 95%). *Note*: Some patients generated more than one complaint, but each complaint was assigned to only one type.

Type IV encounters, defined as flawed or inadequate patient counselling from the pharmacist, were experienced by 15.5% of our sample (*n *= 59). These included complaints about the pharmacy providing wrong information on drug manufacture status and availability (*e.g*., being told a medication had been discontinued), failure to advise about need to refrigerate, and misstatements regarding the government DPS scheme's exclusion of fertility medications. No patient complaint was received involving any pharmacy's pricing of medicine or supplies. From patients who used a C pharmacy, there were an additional 19 unsatisfactory occurrences (5%) that could not be classified (Type V). The pharmacy service complaint type did not vary significantly from 2007 to 2008 (see Figure 3).

## Discussion

The roles and responsibilities of pharmacists are changing rapidly in the current health care environment [4]. Particularly for patients with chronic conditions like diabetes [5], cardiovascular or renal disease [3,6], migraine [7], asthma [8], rheumatoid arthritis [9], or long-term psychiatric illness [10], the patient-pharmacist relationship represents a unique opportunity for improving health delivery and facilitating the work of other providers. While some infertility etiologies have been classified as chronic [11,12], the patient-pharmacist dynamic has not been specifically studied in a reproductive medicine context until now.

The current investigation yielded several unanticipated findings. We found fertility patients in Ireland can be a useful source of direct information about pharmacy performance. These patients, in general, are motivated and have a good level of personal knowledge about their own treatment plan and associated medications. However, while some adverse pharmacy encounters reported by our patients did not reach crisis level, the issues lodged did occasionally identify actual dispensing errors. Pharmacy mistakes of this kind can have disastrous reproductive consequences if uncorrected, as previously reported [13]. In the current sample, C pharmacies were responsible for a disproportionate share of unsatisfactory encounters reported by patients. But interestingly, the most common pharmacy error type did not involve gonadotropins. It was surprising to record the highest level of dispensing error (40.8%) in prescriptions for anti-coagulants, steroids, vitamins, antibiotics or other adjunctive non-fertility agents to our patients.

Since the frequency of service-related issues has not been previously published for any large population of fertility patients, our observed rate of adverse pharmacy encounters (11.8%) could not be benchmarked against other reference groups for comparison. Although this centre's quality management apparatus is designed with a sensitivity threshold to detect problems more subtle than adverse drug events, a large study confined to serious insulin dosing errors during 2000 and 2001 found such problems to be "common" [14].

The second most frequent error type reported by patients dealt with medical equipment or supplies, which represented 20.3% of all adverse pharmacy encounters among our fertility patients (Type III). These complaints typically involved the C pharmacy providing the incorrect gauge needle (or dispensing an insufficient quantity of the proper needle) used for subcutaneous injection of prescribed medication. Patients were also inconvenienced by the unwillingness or inability of C pharmacies to provide biohazard receptacles ("safe sharps bin"), which necessitated the patient calling the IVF clinic for further advice. About 18% of patient complaints alerted us major problems with the gonadotropin prescription itself (Type I). This included incorrect drug being dispensed, label-prescription mismatch, and inappropriate concentration/wrong delivery device. In this group of complaints fertility patients registered substantial worry, perhaps because the gonadotropins were regarded as the most critical ingredient in their fertility treatment sequence.

While these data offer a novel perspective on interactions between fertility patients and their pharmacists, some of our findings should be interpreted with caution. We were only able to evaluate two "specialty" pharmacies used by these study patients, and additional pharmacies of this type should be included in future investigations. It is also important to differentiate an "adverse drug event" from "pharmacy complaint". To be sure, some complaints made by patients were dangerous dispensing errors. Yet the combination of nursing and medical staff surveillance together with fertility patients who asserted active roles in their own medical care intercepted these problems before they escalated to the level of patient injury. Nevertheless, this study confirms the vital role for prevention in strategies to reduce pharmacy error in the delivery of advanced reproductive treatments. Since patients may have been less likely to lodge a complaint against a pharmacy if they selected it themselves, the actual frequency of adverse pharmacy encounters may actually be higher than we observed. Finally, electronic prescribing is not widely used in Ireland and each prescription must be physically brought to the pharmacy. This "hand carrying" of prescriptions may not necessarily have been by the patient herself, and it was not possible to discern which (if any) pharmacy service complaints were initiated by a husband or other family member. Increased use of information technologies in prescribing and dispensing medications should lower the rate of adverse pharmacy encounters and medication errors in Ireland.

How fertility patients select a particular pharmacy is a complex matter, and factors influencing pharmacy choice will differ depending on practice region. Yet, interviews with fertility patients here suggest that convenience is by far the most important consideration. The consistent pattern of Type V complaint regarding S pharmacies observed in this study tends to support this hypothesis. We found some C pharmacies performed exceptionally well and demonstrated a high rate of patient satisfaction, while some S pharmacies did not meet key patient expectations. However, the frequency of unsatisfactory pharmacy encounters was significantly higher when fertility patients did not select an S pharmacy. In Ireland, physicians are not permitted to recommend any pharmacist or retail pharmacy business "otherwise than in the exercise of his or her professional judgment" [15]. Yet the current study brings some important questions into focus both for physicians and pharmacists: What makes a pharmacy designate itself as a "community" or "specialty" pharmacy? Is there a minimum volume of fertility-related prescriptions that a pharmacy should dispense to maintain proficiency in this specific branch of pharmacy practice? Are physicians ethically obligated to guide a fertility patient's choice of pharmacy, considering the profound difference in medication error risk identified in this research? This study, believed to be the first of its kind, brings into sharp relief how fertility patients perceive S and C pharmacies in Ireland differently, and suggests a multidisciplinary approach for further investigations. Differences in fertility patient satisfaction by pharmacy type will be difficult to explain without such study.

## Conclusion

As the number of patients seeking reproductive treatment increases, the number of fertility-related prescriptions will also grow. How these prescriptions are dispensed will continue to be an important issue not just for health economists but clinicians too, in an attempt to demonstrate effectiveness of the patient-pharmacist dynamic [16]. Although the "specialty" vs. "community" pharmacy nomenclature is informal, this is an Irish tradition unlikely to be discontinued. It was outside the scope of our study to define or challenge these designations. The labels did appear to be accurate and provided useful descriptive information about scope, effectiveness and safety of pharmacy practice. However, the substantial difference in fertility patient satisfaction according to pharmacy type warrants further tracking, and forms the basis of ongoing research at our institutions.

## Competing interests

The authors declare that they have no competing interests.

## Authors' contributions

ESS was research consultant in reproductive medicine, SAS was visiting research scientist, DJG, BMGO'C, UER, AOF, PS, and KMB were nurses involved in patient management and pharmacy data collection, LVS was clinical associate, GUM was health services quality manager, DJW was lead physician and medical director who conceived the research and provided general project oversight. All authors read and approved the final manuscript.
